# SHAP-interpretable machine learning integrating exposures and multi-omics reveals immune alterations and biomarkers in COPD–lung cancer comorbidity

**DOI:** 10.3389/fimmu.2026.1875146

**Published:** 2026-08-07

**Authors:** Yiran Fei, Yinying Chai, Shiyuan Tong, Bohao Sun, Ziqiang Chen, Shiliang Chen, Zhezhong Zhang, Yibo He, Shengliang Qiu

**Affiliations:** 1 The First Affiliated Hospital of Zhejiang Chinese Medical University (Zhejiang Provincial Hospital of Chinese Medicine), Hangzhou, Zhejiang, China; 2 State Key Laboratory of Medical Neurobiology and Ministry of Education (MOE) Frontiers Center for Brain Science, Institutes of Brain Science, Fudan University, Shanghai, China; 3 Department of Pathology, Second Affiliated Hospital, School of Medicine, Zhejiang University, Hangzhou, China; 4 Ear, Nose, and Throat (ENT) Institute and Department of Otorhinolaryngology, Eye and ENT Hospital, Fudan University, Shanghai, China; 5 Keqiao Research Institute, Zhejiang Chinese Medical University, Keqiao District, Shaoxing, Zhejiang, China

**Keywords:** artificial intelligence (AI), chronic obstructive pulmonary disease (COPD), immune microenvironment, lung cancer, multi-omics integration

## Abstract

**Introduction:**

Chronic obstructive pulmonary disease (COPD) and lung cancer (LC) frequently co-occur and share environmental and biological determinants, yet their cross-scale associations remain incompletely understood. Artificial intelligence and machine learning–based integration of exposome and multi-omics data provide new opportunities for dissecting this complex comorbidity.

**Methods:**

This study established a sequential, cross-scale framework integrating global epidemiological analysis of GBD data (1990–2021), exposome-wide risk factor assessment using random forest classification with SHAP interpretation, immune microenvironment characterization via CIBERSORT and single-cell RNA sequencing with LLM-assisted annotation, candidate gene prioritization through SMR analysis, LASSO regression, and machine learning classifiers, and experimental validation using RT-qPCR, western blotting, immunohistochemistry, dual immunofluorescence, and macrophage–epithelial Transwell co-culture.

**Results:**

COPD and LC demonstrated persistent global co-occurrence patterns across 204 countries and territories. SHAP analysis identified smoking, particulate matter pollution, and residential radon as major shared risk factors. Bulk and single-cell transcriptomic analyses revealed consistent immune microenvironment remodeling, with macrophages as the predominant shared immune population. Multi-omics intersection and machine learning prioritization identified TREM1 and ODF2L as shared hub genes, significantly downregulated in COPD and LC tissues. Macrophage-specific silencing of TREM1 or ODF2L attenuated macrophage-mediated promotion of A549 cell migration and proliferation, and dual immunofluorescence confirmed macrophage-associated localization of both proteins.

**Discussion:**

These findings provide a cross-scale perspective linking environmental exposures, macrophage-centered immune alterations, and COPD–LC comorbidity. TREM1 and ODF2L represent promising macrophage-associated candidate biomarkers and potential therapeutic targets. Integrating interpretable machine learning with exposome and multi-omics data offers a robust framework for biomarker discovery in chronic respiratory disease comorbidity.

## Introduction

Chronic obstructive pulmonary disease (COPD) and lung cancer (LC) constitute a major source of morbidity and mortality worldwide. COPD is characterized by persistent respiratory symptoms and airflow limitation resulting from airway and/or alveolar abnormalities, typically caused by significant exposure to noxious particles or gases (1). LC represents a heterogeneous group of malignant neoplasms originating from the respiratory epithelium, with non-small cell lung cancer (NSCLC) accounting for the majority of cases (2–4). According to the Global Burden of Disease (GBD) Study 2021, COPD ranks among the leading causes of death globally, while LC remains the most commonly diagnosed respiratory malignancy and the leading cause of cancer-related mortality (5, 6). These diseases frequently coexist in clinical practice, with studies indicating that a substantial proportion of patients diagnosed with LC have concurrent COPD, and reduced lung function serves as an important predictor of LC risk (7). The comorbidity of COPD and LC is associated with substantially worse clinical outcomes compared to either disease alone, including reduced overall survival and increased treatment complexity (8). Despite its significance, COPD remains underdiagnosed and undertreated in patients with LC, with recent reports revealing high prevalence of undiagnosed COPD among patients undergoing LC screening (8). Furthermore, the global burden patterns exhibit marked spatial heterogeneity, with significant disparities between high-income and low-income regions in the relative disease burden, highlighting the need for integrated approaches to understand comorbidity across diverse healthcare settings (9).

The biological mechanisms underlying the COPD-LC relationship center on chronic inflammation and the concept of field cancerization, where persistent inflammatory responses in the respiratory tract create a microenvironment conducive to malignant transformation (7). Chronic exposure to cigarette smoke and other noxious stimuli can induce sustained activation of the NF-κB signaling pathway, leading to the production of pro-inflammatory mediators such as TNF-α, IL-1β, and IL-6, thereby perpetuating airway inflammation and contributing to carcinogenesis. In addition, IL-6-mediated activation of the STAT3 pathway has been implicated in both chronic inflammation and tumor-promoting processes in the lung (10, 11). This process involves complex interactions between environmental exposures and immune dysregulation, characterized by the accumulation and functional alteration of innate and adaptive immune cells (7). Macrophages, in particular, play a pivotal role in maintaining chronic inflammation and promoting tissue remodeling in both diseases, with M2-polarized macrophages contributing to immunosuppressive microenvironments that support tumorigenesis (12). The tumor microenvironment creates unequal distributions of oxygen and nutrients that further influence macrophage polarization, with hypoxic areas promoting M2-like phenotypes that drive tumor progression (13). Furthermore, while smoking and ambient air pollution are established shared risk factors, the exposome-wide contributions to COPD-LC comorbidity, including climate factors, occupational hazards, and residential exposures, have been increasingly recognized as critical components of disease etiology (14). Collectively, these observations support a unifying biological hypothesis that shared environmental exposures may initiate persistent immune remodeling within the lung microenvironment, with macrophages acting as key mediators linking chronic inflammation, tissue remodeling, and malignant transformation. Because macrophages are the primary innate immune cells responsible for sensing and responding to inhaled toxicants, prolonged environmental exposure may alter their abundance, activation state, and intercellular communication, thereby establishing common molecular pathways that contribute to COPD-LC comorbidity (15).

Recent advances in multi-omics technologies and artificial intelligence offer opportunities to dissect these complex disease mechanisms (16). Single-cell RNA sequencing (scRNA-seq) has enabled insights into cellular heterogeneity and immune microenvironment remodeling at single-cell resolution, as demonstrated in comprehensive atlases of LC and chronic lung diseases (17). Machine learning approaches integrating transcriptomic and genetic data have shown improved prediction performance for disease outcomes in cancer genomics studies (18). However, effective strategies for harmonizing exposome and multi-omics data types and extracting clinically meaningful insights remain underdeveloped (19). Mendelian randomization (MR) approaches provide methods to establish causal relationships between molecular features and disease outcomes, with recent druggable genome-wide analyses identifying novel therapeutic targets for LC through integration of Genome-Wide Association Study (GWAS) and quantitative trait loci data (20). Interpretable artificial intelligence methods, such as SHapley Additive exPlanations (SHAP), have been applied to identify key biomarkers and risk factors in cancer genomics and immunology, demonstrating their potential to uncover latent biological patterns (21); however, their systematic application to chronic respiratory disease comorbidity remains limited.

**Figure 1:**
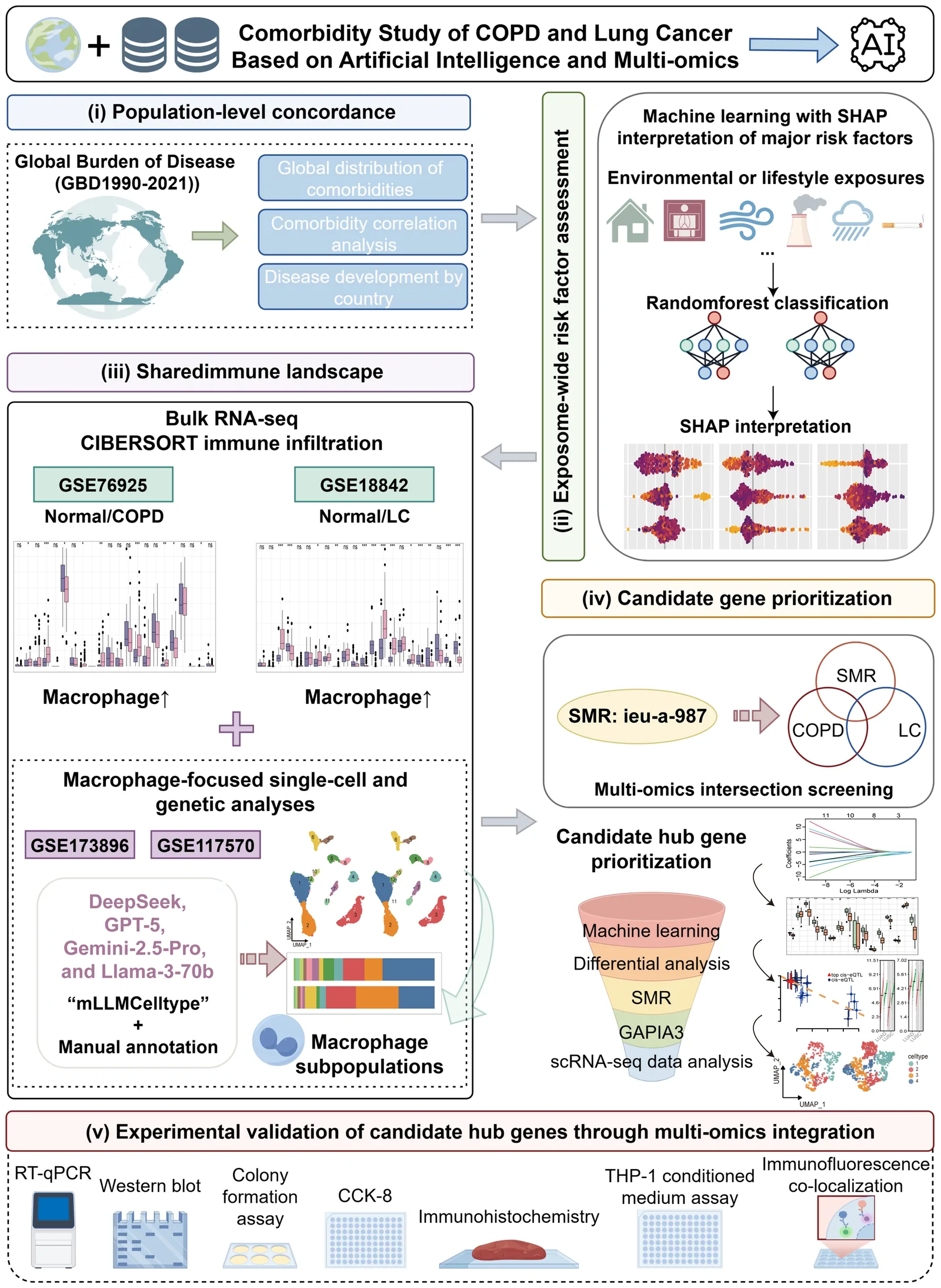
Study workflow for the integrated analysis of COPD and LC comorbidity. This study established a stepwise workflow to investigate COPD–LC comorbidity from population-level observations to molecular mechanisms and experimental validation. (1) The global epidemiological landscape of COPD and LC was first characterized using Global Burden of Disease (GBD) 1990–2021 data, including spatial distribution, temporal trends, and assessment of concordant disease-burden patterns across 204 countries and territories. (2) To explore potential environmental determinants underlying these population-level patterns, exposome-wide risk factors were evaluated using random forest classification, and SHAP analysis was applied to quantify the contribution of individual environmental and lifestyle exposures. (3) To investigate whether exposure-associated COPD–LC patterns are accompanied by shared biological alterations, bulk transcriptomic datasets were analyzed using CIBERSORT-based immune infiltration profiling, followed by single-cell RNA sequencing with LLM-assisted annotation and manual curation to resolve cellular heterogeneity and identify key immune cell populations, particularly macrophages. (4) Guided by the macrophage-related findings, candidate genes were prioritized by integrating COPD and LC macrophage differentially expressed genes with lung cancer SMR-supported genes. The resulting candidates were further evaluated through intersection analysis, LASSO regression, multiple machine-learning classifiers, independent GEO validation cohorts, GEPIA3 analysis, and macrophage subtype-specific expression analysis to identify robust hub genes. (5) Finally, the functional relevance of the prioritized genes, TREM1 and ODF2L, was validated using clinical tissue specimens and *in vitro* models, including RT-qPCR, western blotting, immunohistochemistry, dual immunofluorescence staining, gene silencing, and macrophage–epithelial co-culture assays.

We therefore designed a sequential, cross-scale framework to investigate the mechanisms underlying COPD–lung cancer (LC) comorbidity. First, we used global epidemiological data to determine whether COPD and LC exhibit reproducible spatial and temporal concordance across countries and regions. Second, random forest modeling with SHAP interpretation was applied to identify environmental factors associated with distinct COPD–LC burden patterns. Third, because environmental exposures are known to drive chronic pulmonary inflammation, we investigated the shared immune microenvironment using immune infiltration analysis and single-cell RNA sequencing to identify the immune cell populations potentially linking COPD and LC. Fourth, because macrophages were consistently identified as the predominant shared immune population, we further investigated whether macrophage-associated molecular alterations could provide a mechanistic link between the two diseases. Macrophage-specific transcriptional signatures were integrated with SMR analysis and machine-learning approaches to prioritize candidate genes supported by complementary cellular and genetic evidence, followed by independent validation in public datasets. Finally, the prioritized genes were further validated using clinical specimens and *in vitro* experiments to evaluate their expression, cellular localization, and biological functions. This sequential framework enabled us to move from population-level observations to immune-cell mechanisms and experimentally validated molecular candidates (**Figure 1**).

## Materials and methods

### 2.1 Analytical workflow

This study followed a sequential analytical workflow in which each stage addressed a distinct research question. Global epidemiological analyses established the population-level concordance between COPD and lung cancer. Machine-learning models were subsequently applied to identify environmental factors associated with different disease-burden patterns. Immune infiltration analysis and single-cell RNA sequencing were then used to characterize the shared immune microenvironment and identify the principal cellular populations involved. Finally, candidate genes were prioritized by integrating macrophage-specific transcriptional signatures, genetic evidence, and machine-learning evaluation, followed by experimental validation.

### 2.2 Data sources and ethical approval

In this study, the global age-standardized incidence rates (ASIRs) and risk factor exposure data for COPD and LC from 1990 to 2021 were extracted from the GBD database (http://ghdx.healthdata.org/gbd-results-tool). Transcriptomic data were obtained from the Gene Expression Omnibus (GEO) database (https://www.ncbi.nlm.nih.gov/geo/). Specifically, bulk RNA-sequencing (RNA-seq) datasets GSE76925 and GSE18842 were utilized to evaluate immune infiltration and served as the training sets for the machine learning diagnostic models of COPD and LC, respectively. Datasets GSE57148 and GSE74706 were downloaded as independent test sets. For scRNA-seq analysis, the GSE173896 (COPD) and GSE117570 (LC) datasets were acquired. GWAS summary data for LC were downloaded from the IEU OpenGWAS project (https://gwas.mrcieu.ac.uk/; GWAS ID: ieu-a-987).

A total of 40 human lung tissue specimens were enrolled in this study and collected from the First Affiliated Hospital of Zhejiang Chinese Medical University (Zhejiang Provincial Hospital of Chinese Medicine). The samples were divided into three groups: normal control lung tissues (n = 20), COPD patient lung tissues (n = 10), and LC tumor tissues (n = 10). Normal lung tissues were obtained from adjacent histologically normal tissues (located > 5 cm from the tumor margin) during LC surgical resection or from patients with benign lung lesions. This study strictly complied with the Declaration of Helsinki. The collection of all tissue samples was approved in advance by the Ethics Committee of the First Affiliated Hospital of Zhejiang Chinese Medical University (Approval No. 2026-KL-197-01).

### 2.3 Global comorbidity patterns and spatial correlation analysis

To quantify and compare regional disease burdens, quartiles of COPD and LC incidence rates across 204 countries and territories were calculated. Based on these thresholds, countries were categorized into four strata: low, lower-middle, upper-middle, and high. By integrating the strata of both diseases, three comorbidity patterns were defined: consistent, COPD-dominant, and LC-dominant patterns (22). Spatial correlations were assessed using Spearman rank correlation coefficients with corresponding confidence intervals across multiple years. A comorbidity severity scoring system (range: 1–4) was further developed, assigning scores of 4 for double-high (critical), 3 for high and upper-middle (severe), 2.5 for double upper-middle, and 2 or 1 for moderate and low levels, respectively. Based on these scores, temporal trends from 1990 to 2021 were compared across developed countries, emerging economies, least developed countries, and other developing regions. Finally, Gaussian similarity was applied to construct network associations among the top 50 high-burden countries, followed by clustering and network visualization using the Louvain community detection algorithm.

### 2.4 Evaluation of risk factors based on machine learning and SHAP interpretation

To quantitatively evaluate the driving effects of various environmental and lifestyle factors on the three comorbidity patterns (target variable y), exposure characteristics of multiple risk factors (independent variables x) across different regions in 2021 were extracted to construct a multi-class prediction model. A random forest model was trained using the randomForest R package (version 4.7-1.2) (23). Given the lack of intuitive interpretability of black-box machine learning models, the kernelshap (version 0.9.0) (24) and shapviz (version 0.10.2) (25) algorithms were introduced to calculate SHAP values. By generating SHAP bee swarm plots, the characteristic contributions and directional impacts (positive or negative) of core risk factors within the consistent, COPD-dominant, and LC-dominant patterns were visualized. Specifically, particulate matter pollution, smoking, and secondhand smoke were shared risk factors for both COPD and LC; high temperature, low temperature, and occupational particulate matter, gases, and fumes were COPD-related factors; and residential radon, occupational carcinogens, and a diet low in fruits were LC-related factors. Ultimately, the global distribution characteristics of these core risk factors were displayed via spatial maps. Positive SHAP values indicate that an exposure increases the probability of predicting a specific COPD–LC comorbidity pattern, whereas negative values indicate the opposite. Importantly, SHAP values quantify the contribution of a feature to model prediction and should not be interpreted as evidence of causality.

### 2.5 Bulk RNA-seq immune infiltration analysis

Based on the GSE76925 (COPD: 111; Control: 40) and GSE18842 (LC: 46; Control: 45) transcriptomic datasets, the immune microenvironment characteristics of diseased and healthy lung tissues were evaluated. Prior to inferring immune cell fractions, the gene expression matrices were preprocessed using the limma R package (version 3.56.2) (26). Subsequently, relying on the CIBERSORT deconvolution algorithm integrated within the IOBR R package (version 0.99.9) (27, 28), the relative infiltration proportions of various immune cell subpopulations in the samples were calculated. To ensure the reliability of the deconvolution results, only samples with a CIBERSORT output of P < 0.05 were retained for subsequent statistical analyses. The composition of immune cell populations at the sample level was visualized using stacked bar plots, and differences in the relative abundance of 22 immune cell types between disease and control groups were compared using boxplots. Statistical significance was assessed using the Wilcoxon rank-sum test.

### 2.6 Processing of scRNA-seq data and AI-assisted annotation

The Seurat R package (version 4.4.0) (29–33) was employed for the initial processing and quality control of the scRNA-seq expression matrices (GSE173896 and GSE117570). During the creation of the Seurat objects, genes expressed in at least 3 cells and cells expressing a minimum of 300 genes were retained. To filter out low-quality cells, dead cells, and doublets, stringent quality control criteria were applied: cells with a detected gene count (nFeature_RNA) between 300 and 2,500 and a mitochondrial gene expression ratio (percent.mt) ≤ 20% were preserved. Subsequently, the top 2,000 highly variable genes (HVGs) were identified, and principal component analysis (PCA) was performed based on these 2,000 HVGs to achieve linear dimensionality reduction. To eliminate batch effects arising from different sample origins (orig.ident), the harmony R package (version 1.0.3) (34) was introduced to integrate multiple samples post-PCA. Based on the corrected Harmony embeddings, principal components were extracted, and cell neighbor graphs were constructed and unsupervised clustering was performed using the FindNeighbors and FindClusters functions. The Uniform Manifold Approximation and Projection (UMAP) algorithm was then applied to visualize the clustering results in a two-dimensional space.

During the cell subpopulation annotation phase, this study adopted a strategy combining large language model (LLM)-assisted evaluation with manual curation. Initially, the mLLMCelltype tool (version 1.3.4) (35) was utilized to invoke multiple advanced LLMs (including DeepSeek, GPT-5, Gemini-2.5-Pro, and Llama-3-70b) for preliminary consensus annotation. For each cluster, the top 10 upregulated markers (log fold change > 0.25, expressed in ≥25% of cells) were submitted. Consensus proportion was defined as the fraction of models returning the identical label; a threshold of ≥0.6 (≥3 of 5 models) and Shannon entropy ≤1.0 were required. Clusters failing these criteria were flagged for manual curation. Final annotation integrated canonical marker genes with manual verification (36). Finally, integrating known canonical cell marker genes, manual verification and curation were conducted to definitively identify and plot the UMAP landscapes and proportional bar charts of the major cell types.

### 2.7 SMR and multi-omics intersection analysis

To explore the potential shared genetic and cellular mechanisms underlying COPD and LC, this study focused on the macrophage populations within the pulmonary microenvironment. Macrophage-specific differentially expressed genes (DEGs) were extracted from the scRNA-seq datasets of COPD and LC, respectively. Concurrently, a SMR analysis was performed using the LC GWAS summary data (ieu-a-987) to incorporate genetic evidence and identify instrumental variable genes with significant causal associations with LC. A Venn diagram was utilized to intersect the SMR-identified genes, COPD macrophage DEGs, and LC macrophage DEGs, and following filtration processing, the overlapping hub genes were ultimately pinpointed.

### 2.8 Construction and evaluation of machine learning diagnostic models

To comprehensively evaluate the diagnostic efficacy of the identified hub genes for the diseases, a multi-algorithm machine learning diagnostic workflow was constructed. First, the expression matrices of candidate genes were extracted from the training sets (GSE76925 and GSE18842), and univariate logistic regression analysis was performed for initial gene screening, retaining genes significantly associated with the disease state (P < 0.05). Subsequently, Least Absolute Shrinkage and Selection Operator (LASSO) regression was applied for further feature selection using the glmnet R package (version 4.1-8) (37–39). The LASSO analysis employed 10-fold cross-validation and determined the optimal penalty coefficient based on the minimum deviance criterion (lambda.min) to minimize the risk of model overfitting and lock in the final feature genes. After determining the feature genes, their expression levels in the training sets and independent test sets (GSE57148 and GSE74706) were subjected to Z-score standardization to eliminate dimensional differences. Following this, relying on the mlr3verse machine learning framework (version 0.3.1) (40), seven distinct classification algorithm models were constructed, including logistic regression (log_reg), linear discriminant analysis (lda), support vector machine (svm), naive Bayes (naive_bayes), k-nearest neighbors (kknn), decision tree (rpart), and random forest. To obtain unbiased performance estimates while preventing overfitting, we employed stratified nested cross-validation: an outer 5-fold cross-validation repeated 10 times estimated generalization performance, while an inner 3-fold loop performed feature selection and hyperparameter tuning. Z-score standardization parameters were derived from training folds only and applied to validation folds to prevent data leakage. Models were compared using mean AUC, sensitivity, and specificity across outer folds. The best-performing model was then trained on the full training set and evaluated on independent external test sets (GSE57148 and GSE74706), with ROC curves plotted to assess diagnostic generalization (41).

### 2.9 Multi-omics validation of TREM1 and ODF2L

Furthermore, the differential expression of the core genes TREM1 and ODF2L was not only confirmed in the transcriptomic data (GSE76925 and GSE18842) but also validated within the machine learning diagnostic models. We further conducted cross-validation across different LC histological subtypes via the GEPIA3 database (https://gepia3.bioinfoliu.com/; TCGA RNA-seq data) to substantiate their biological and clinical relevance, noting that TCGA and GEO datasets differ in cohort composition and sequencing protocols. To deeply investigate the intra-population heterogeneity of macrophages, the subset function was used to extract all macrophage subpopulations from Section 2.6 for secondary dimensionality reduction and clustering. The extracted subset underwent re-identification of highly variable genes, data scaling, PCA dimensionality reduction, and Harmony batch effect correction. Principal components were selected for UMAP visualization and unsupervised clustering. Macrophages were further re-clustered and annotated into four macrophage subpopulations based on established marker genes, including inflammatory macrophages, resident alveolar macrophages, monocyte-derived macrophages, and tumor-associated macrophages. The expression patterns of TREM1 and ODF2L were subsequently mapped across these annotated macrophage subsets.

### 2.10 Cell culture

Human normal bronchial epithelial cells (BEAS-2B), lung adenocarcinoma cells (A549), and monocytic cells (THP-1) were obtained from the BeNa Culture Collection (Henan, China). BEAS-2B and A549 cells were cultured in DMEM supplemented with 10% fetal bovine serum (FBS), while THP-1 cells were maintained in RPMI-1640 medium containing 10% FBS. All cells were incubated at 37 °C in a humidified atmosphere with 5% CO_2_. THP-1 cells were differentiated into macrophage-like cells using phorbol 12-myristate 13-acetate (PMA) at a final concentration of 100 ng/mL for 24–48 h. After differentiation, cells were washed with PBS and cultured in fresh medium for subsequent experiments.

### 2.11 RT-qPCR

Total RNA was extracted from cells via the TRIzol method: homogenize cells, add TRIzol reagent for lysis (room temperature, 5 min), then chloroform. After centrifugation, transfer the upper aqueous phase (containing RNA) to an RNase-free tube, add equal-volume isopropanol for precipitation, and discard the supernatant post-centrifugation. Rinse the RNA pellet with 75% ethanol, air-dry for 10 min at room temperature, and dissolve for use. After verifying RNA concentration and purity, synthesize complementary DNA (cDNA) via a reverse transcription kit. RT-qPCR was performed on a QuantStudio 6 Flex System (Life Technologies) using EnTurbo™ SYBR Green PCR SuperMix (ELK Biotechnology, China) with triplicate wells per sample. Relative expression levels of TREM1 and ODF2L were normalized to GAPDH and calculated by the 2^−ΔΔCt method. Primer sequences are listed in **Supplementary Table 1**.

### 2.12 Western blot analysis

Total protein was extracted from cultured cells using RIPA lysis buffer supplemented with protease and phosphatase inhibitors, and protein concentrations were determined using a BCA Protein Assay Kit (ELK Biotechnology, China). Equal amounts of protein were separated by SDS-PAGE and transferred onto polyvinylidene fluoride (PVDF) membranes. After blocking with 5% non-fat milk for 1 h at room temperature, the membranes were incubated overnight at 4 °C with primary antibodies against TREM1, ODF2L, phosphorylated NF-κB p65 (p-p65), total NF-κB p65 (p65), and GAPDH. After washing with Tris-buffered saline containing 0.1% Tween-20 (TBST), the membranes were incubated with horseradish peroxidase (HRP)-conjugated secondary antibodies for 1 h at room temperature. Protein bands were visualized using an enhanced chemiluminescence (ECL) detection system and quantified using ImageJ software. The expression levels of TREM1 and ODF2L were normalized to GAPDH, whereas NF-κB activation was evaluated by calculating the p-p65/p65 ratio. Antibody details are provided in **Supplementary Table 2**.

### 2.13 Cell transfection

A549 cells were transiently transfected with small interfering RNAs (siRNAs) targeting TREM1 (si-TREM1), ODF2L (si-ODF2L), or a negative control siRNA (si-NC) using Lipofectamine 3000 according to the manufacturer’s instructions. For macrophage-based functional experiments, THP-1-derived macrophages were transfected with short hairpin RNAs (shRNAs) targeting TREM1 (shTREM1–1 and shTREM1-2), ODF2L (shODF2L-1 and shODF2L-2), or a non-targeting control shRNA (shNC). Cells were transfected at approximately 60–70% confluence and cultured under standard conditions before subsequent experiments. Knockdown efficiency was confirmed by RT-qPCR and/or western blot analysis. The sequences of siRNAs and shRNAs are provided in **Supplementary Table 3**.

### 2.14 Cell proliferation assay (CCK-8)

Cell proliferation was assessed using the Cell Counting Kit-8 (CCK-8; ELK Biotechnology, China) according to the manufacturer’s instructions. For A549 cell intrinsic functional assays, siRNA-transfected A549 cells were seeded into 96-well plates at an appropriate density. At the indicated time points (24, 48, 72, 96, and 120 h after seeding), 10 μL of CCK-8 solution was added to each well and incubated at 37 °C for 2 h. Absorbance was measured at 450 nm using a microplate reader.

### 2.15 Colony formation assay

Cell colony formation ability was assessed using a colony formation assay. Transfected A549 cells were seeded into 6-well plates at a low density of approximately 500–1000 cells per well and cultured under standard conditions for 10–14 days to allow colony formation. The culture medium was renewed every 3–4 days. At the end of incubation, cells were fixed with 4% paraformaldehyde for 15 min and stained with 0.1% crystal violet solution for 20 min. Following rinsing with phosphate-buffered saline (PBS) and air-drying, visible colonies containing more than 50 cells were manually counted.

### 2.16 Immunohistochemical staining and semi-quantitative evaluation

Resected pulmonary tissues were fixed in 4% paraformaldehyde, paraffin-embedded, and sectioned at 4 μm. After deparaffinization and rehydration, antigen retrieval was performed using EDTA buffer, followed by blocking of endogenous peroxidase activity with 3% H_2_O_2_ and nonspecific binding with 5% BSA. Sections were incubated overnight at 4 °C with anti-TREM1 antibody (rabbit polyclonal, abinScience, China) and anti-ODF2L antibody (rabbit polyclonal, Immunoway, China), followed by incubation with HRP-conjugated secondary antibodies. Immunoreactivity was visualized using DAB, and nuclei were counterstained with hematoxylin. Staining intensity was semi-quantitatively scored as 0 (negative), 1 (weak, light yellow), 2 (moderate, brownish-yellow), and 3 (strong, dark brown). The average staining intensity score for each group was calculated, and intergroup differences were statistically compared.

### 2.17 Indirect macrophage–epithelial Transwell co-culture

THP-1-derived macrophages were transfected with shRNAs targeting TREM1, ODF2L, or a non-targeting control shRNA (shNC). Knockdown efficiency was confirmed by western blot analysis. Transfected macrophages were seeded into the upper chamber of Transwell inserts, while A549 cells were cultured in the lower chamber to establish an indirect macrophage–epithelial co-culture system. After co-culture, A549 cell migration was evaluated using Transwell migration assays, and cell proliferation was assessed using the CCK-8 assay. Protein lysates from THP-1-derived macrophages were collected for western blot analysis of TREM1, ODF2L, p-p65, p65, and GAPDH.

### 2.18 Dual immunofluorescence staining

Paraffin-embedded human lung tissue sections were deparaffinized, rehydrated, and subjected to antigen retrieval. After blocking with normal serum, sections were incubated overnight at 4 °C with primary antibodies against TREM1 or ODF2L, together with the macrophage marker CD68. After washing, sections were incubated with species-specific fluorescent secondary antibodies. Nuclei were counterstained with DAPI. Representative merged and single-channel images were obtained at low magnification, and selected regions of interest were further examined at higher magnification (original magnification, ×40). Relative fluorescence intensities of TREM1 or ODF2L and CD68 were quantified using ImageJ software. Line profile analysis was performed using the Plot Profile function in ImageJ to evaluate the spatial distribution of TREM1–CD68 or ODF2L–CD68 fluorescence signals.

### 2.19 Statistical analysis

All statistical analyses and graphical visualizations of the data were performed using R software (version 4.4.1) and GraphPad Prism (version 9.5). For continuous variables, Student’s t-test or the Wilcoxon rank-sum test was applied according to data distribution, while one-way ANOVA was used for comparisons among multiple groups. Correlation analyses were conducted using the Spearman rank correlation coefficient. A two-sided P value < 0.05 was considered statistically significant.

## Results

### 3.1 Global concordance of COPD and LC

**Figure 2:**
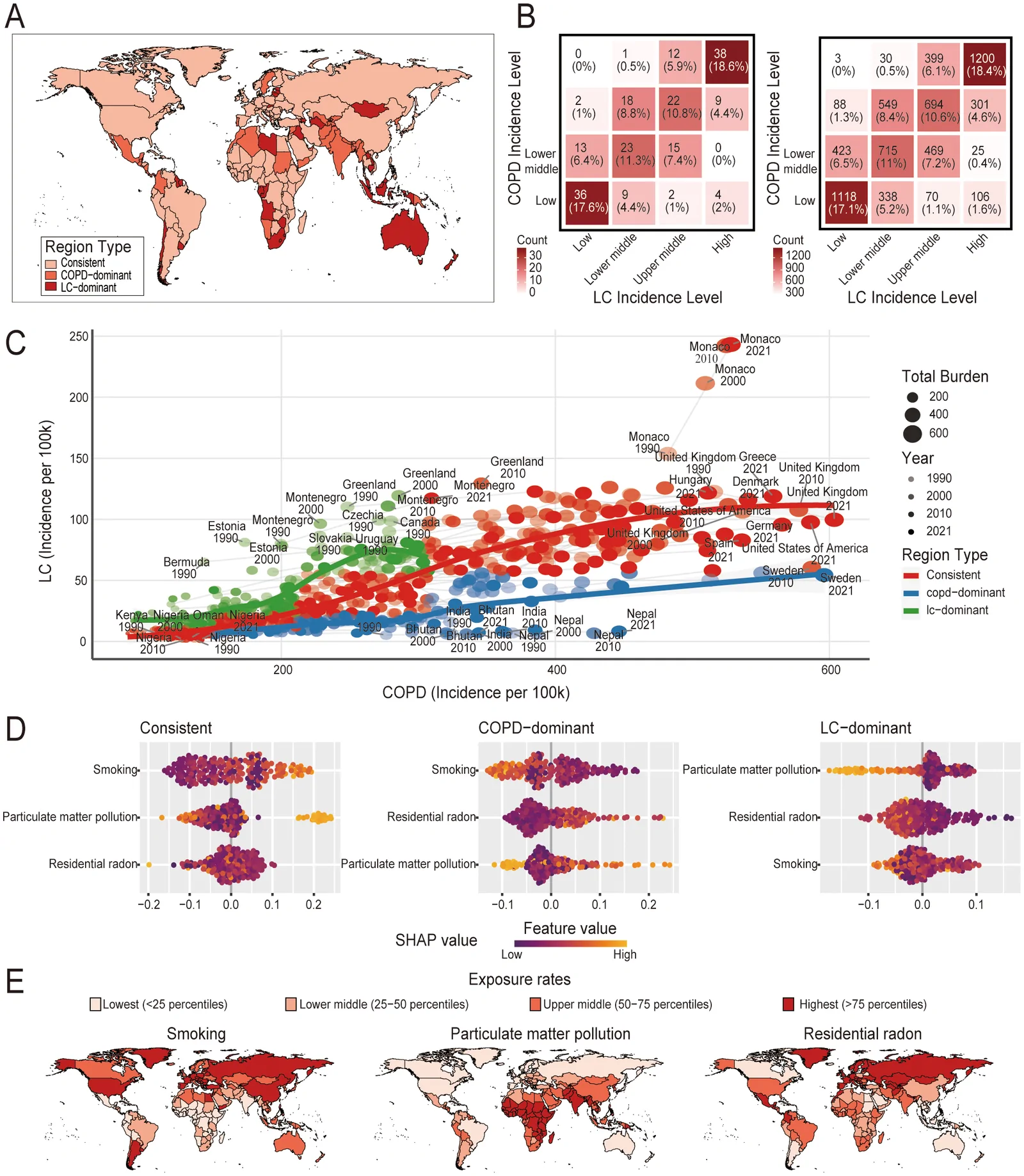
Global epidemiological patterns and environmental drivers of COPD–LC comorbidity. **(A)** Global distribution of COPD–LC comorbidity patterns in 2021, including the consistent, COPD-dominant, and LC-dominant patterns. **(B)** Heatmaps showing the distribution of countries and regions stratified by COPD and LC incidence levels in 2021 (left) and across the period from 1990 to 2021 (right). **(C)** Scatter plots showing the correlation between COPD and LC incidence across countries and regions in 1990, 2000, 2010, and 2021. Bubble size represents the overall disease burden, and colors indicate different comorbidity patterns. **(D)** SHAP summary plots showing the relative contributions of the major environmental risk factors in the consistent, COPD-dominant, and LC-dominant patterns. Each point represents one country or region; the x-axis indicates the SHAP value, and colors denote the feature value from low to high. **(E)** Global exposure distributions of the three representative environmental risk factors identified by SHAP analysis, including smoking, particulate matter pollution, and residential radon.

In 2021, the age-standardized incidence rates (ASIRs) of both COPD and LC exhibited marked global spatial heterogeneity (**Supplementary Figures 1A, B**). COPD showed a broader geographical distribution of high-incidence regions, whereas LC incidence was more concentrated in Europe and other high-burden regions. Further classification of countries and territories according to the relative burden of COPD and LC demonstrated that the consistent comorbidity pattern accounted for the largest proportion worldwide, whereas the COPD-dominant and LC-dominant patterns were less common and displayed regional clustering (**Figure 2A**). Concordance analysis further supported this global association. In 2021, 58.3% (119/204) of countries and territories were classified into concordant COPD and LC incidence strata, with the high-high (38/204, 18.6%) and low-low (36/204, 17.6%) categories representing the largest proportions, followed by the lower middle-lower middle (23/204, 11.3%) and upper middle-upper middle (22/204, 10.8%) categories (**Figure 2B**). This concordance remained stable throughout 1990–2021, during which 57.1% (3727/6528) of all country-year observations were assigned to concordant incidence strata, whereas highly discordant combinations remained relatively uncommon. Scatter plot analysis further demonstrated a persistent positive association between COPD and LC incidence across countries and territories over time (**Figure 2C**). Spearman correlation analysis stratified by comorbidity pattern showed that the strongest correlation was observed in the consistent pattern (rho = 0.938, P < 0.001, n = 3727), followed by the LC-dominant pattern (rho = 0.838, P < 0.001, n = 1492), whereas the COPD-dominant pattern also exhibited a significant positive correlation (rho = 0.544, P < 0.001, n = 1309). Countries belonging to the consistent pattern were distributed along the principal positive correlation trajectory, whereas COPD-dominant and LC-dominant countries deviated below and above this trend, respectively. Temporal analyses across different development categories further demonstrated that these global comorbidity patterns remained stable throughout the study period despite regional differences in overall disease burden. Consistent with this observation, country similarity analysis showed that countries with comparable epidemiological profiles clustered together, further supporting the robustness and persistence of the global concordance between COPD and LC (**Supplementary Figures 1C–G**).

### 3.2 Machine learning identifies major environmental drivers of COPD–LC comorbidity

An interpretable machine-learning model combined with SHapley Additive exPlanations (SHAP) was applied to evaluate the relative importance of environmental exposures across different COPD–LC comorbidity patterns (**Figure 2D**). Higher absolute SHAP values indicate greater contributions of individual exposures to model prediction. The SHAP analysis consistently identified smoking, particulate matter pollution, and residential radon as the three most important environmental factors across the different COPD–LC comorbidity patterns (**Figure 2D**). In the consistent pattern, smoking ranked as the strongest predictor, followed by particulate matter pollution and residential radon. In the COPD-dominant pattern, smoking remained the leading contributor, whereas residential radon and particulate matter pollution ranked second and third, respectively. By contrast, in the LC-dominant pattern, residential radon became the highest-ranked predictor, followed by particulate matter pollution and smoking.

To further illustrate the spatial distribution of these representative exposures, their global exposure patterns are shown in **Figure 2E**. Smoking exhibited relatively high exposure levels across Europe, East Asia, and several regions of North America, whereas particulate matter pollution was predominantly concentrated in South Asia, the Middle East, and large parts of Africa. Residential radon showed higher exposure levels in North America, northern Europe, and northern Asia. The spatial distributions of the remaining environmental exposures are provided in the Supplementary Material (**Supplementary Figure 2**). Having identified environmental factors associated with different COPD–LC burden patterns, and considering that chronic exposure-related inflammation may represent a biological link between environmental insults and respiratory disease progression, we next investigated whether COPD and LC shared common alterations in the pulmonary immune microenvironment.

### 3.3 Immune infiltration and single-cell landscape analyses

**Figure 3:**
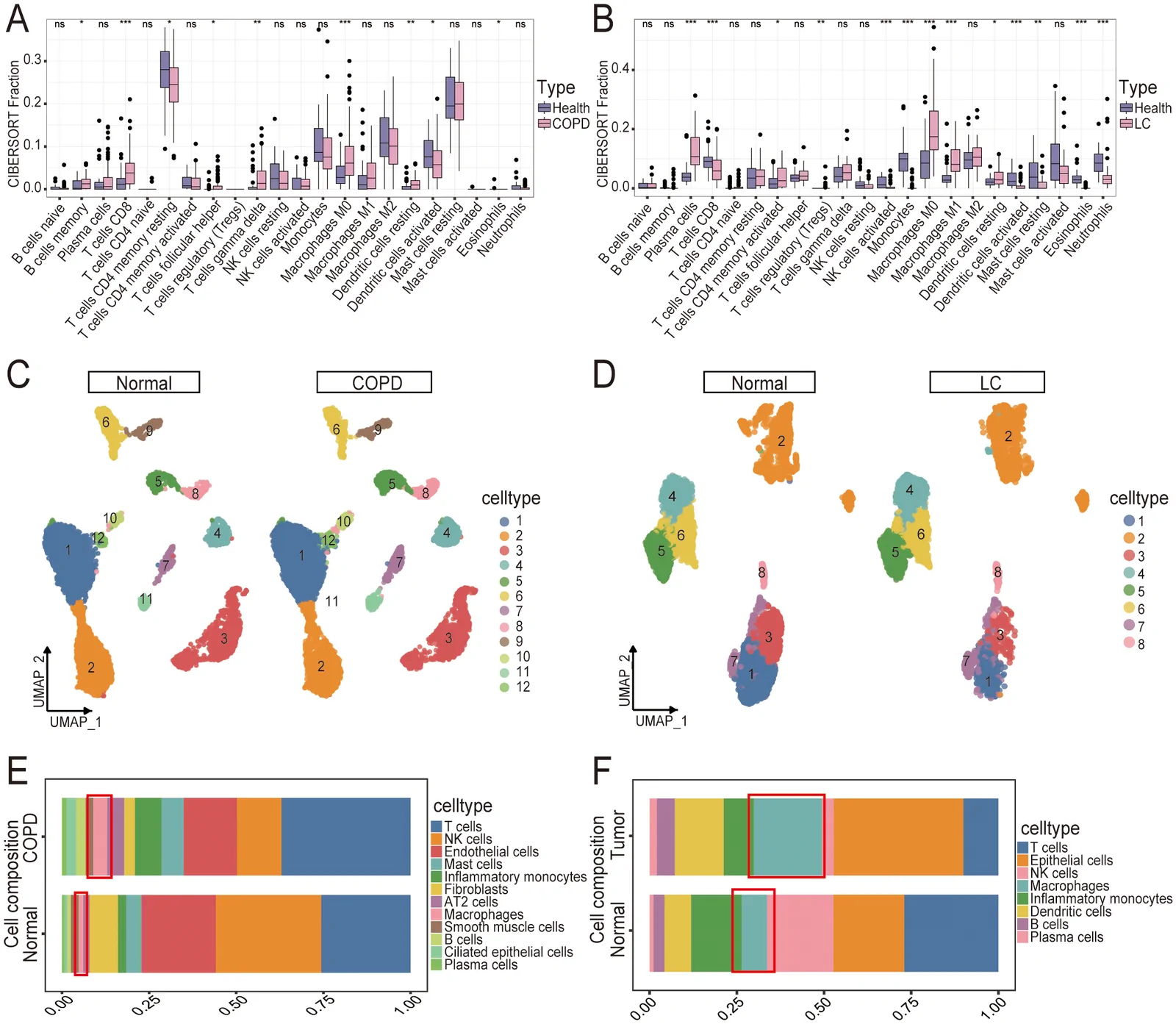
Immune infiltration and single-cell landscape analyses in COPD and LC. **(A, B)** Comparison of immune cell infiltration fractions between healthy controls and COPD samples **(A)** or LC samples **(B)**, as estimated by CIBERSORT (GSE76925 and GSE18842, respectively). Statistical significance is indicated as ***P < 0.001, **P < 0.01, *P < 0.05, and ns, not significant. **(C–F)** Single-cell transcriptomic landscapes of COPD and LC. UMAP plots show the distribution of cell clusters in normal and COPD lung tissues **(C)** and in normal and LC tissues **(D)**, based on GSE173896 and GSE117570, respectively. Cell composition plots show the relative proportions of annotated cell populations in normal versus COPD samples **(E)** and in normal versus LC samples **(F)**.

Immune infiltration analysis was performed using the COPD dataset GSE76925 and the LC dataset GSE18842. Compared with healthy controls, both COPD and LC exhibited substantial immune microenvironment remodeling (**Figures 3A, B**). Notably, macrophage-associated populations were consistently enriched in both diseases. In COPD, M0 macrophages showed a significant increase, accompanied by alterations in CD8^+^ T cells, γδ T cells, and resting dendritic cells (**Figure 3A**). Similarly, LC tissues demonstrated significantly increased infiltration of both M0 and M1 macrophages, together with changes in plasma cells, CD8^+^ T cells, activated natural killer (NK) cells, monocytes, activated dendritic cells, eosinophils, and neutrophils (**Figure 3B**). These findings prompted us to further investigate macrophage heterogeneity at single-cell resolution.

To further characterize the cellular landscape, single-cell RNA sequencing datasets GSE173896 (COPD) and GSE117570 (LC) were analyzed. After quality control, unsupervised clustering and cell-type annotation identified the major immune and stromal cell populations in both datasets (**Figures 3C, D**), whereas the detailed cell annotation strategy and marker genes are provided in the Supplementary Material (**Supplementary Figure 3**; **Supplementary Table 4**). Analysis of cell-type composition further supported the bulk immune infiltration results. Macrophages remained one of the predominant immune cell populations in COPD tissues and were markedly enriched in LC tissues compared with their corresponding controls (**Figures 3E, F**), whereas additional changes were observed in T cells, NK cells, epithelial cells, and inflammatory monocytes.

**Figure 4:**
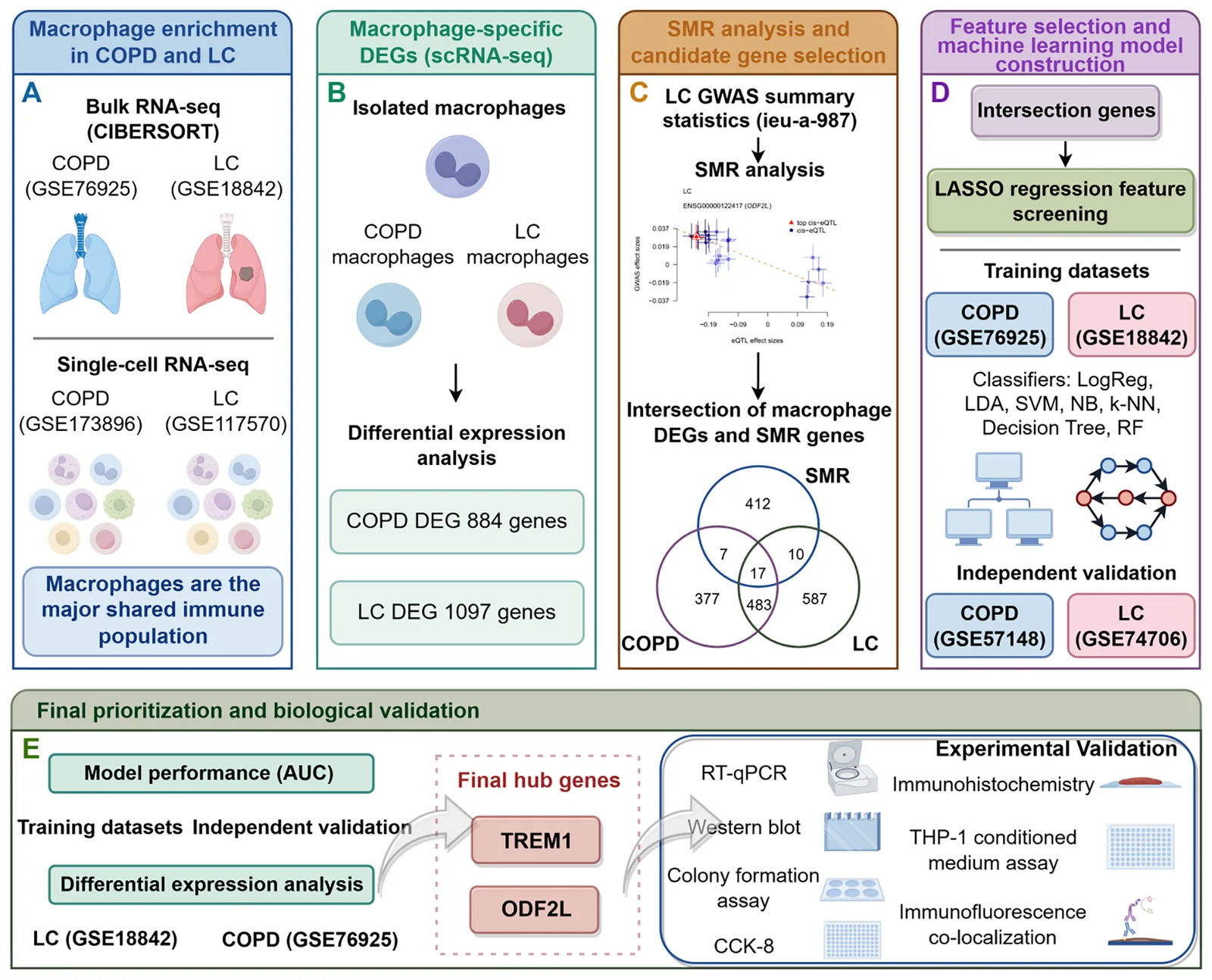
Workflow for hub gene prioritization through integrative multi-omics analysis. **(A)** Bulk transcriptomic immune infiltration and single-cell RNA sequencing analyses identified macrophages as the major shared immune population in COPD and LC. **(B)** Macrophage-specific differentially expressed genes (DEGs) were identified from the single-cell datasets. **(C)** Macrophage DEGs were integrated with genes prioritized by summary data-based Mendelian randomization (SMR) analysis to obtain candidate genes. **(D)** Candidate genes were subjected to LASSO feature selection and machine-learning model construction with independent validation. **(E)** Model evaluation, differential expression analyses, and experimental validation led to the prioritization of TREM1 and ODF2L as the final hub genes.

Because macrophages were consistently identified as the predominant shared immune population by both bulk transcriptomic and single-cell analyses, we hypothesized that macrophage-associated molecular alterations may contribute to the shared pathogenesis of COPD and LC. Therefore, subsequent analyses focused on macrophage-specific candidate genes. Differentially expressed genes identified within macrophages were therefore integrated with SMR and machine-learning analyses to prioritize candidate hub genes underlying COPD–LC comorbidity. The overall workflow for hub gene prioritization and experimental validation is summarized in **Figure 4**.

### 3.4 Multi-omics and machine learning analysis of shared hub genes

**Figure 5:**
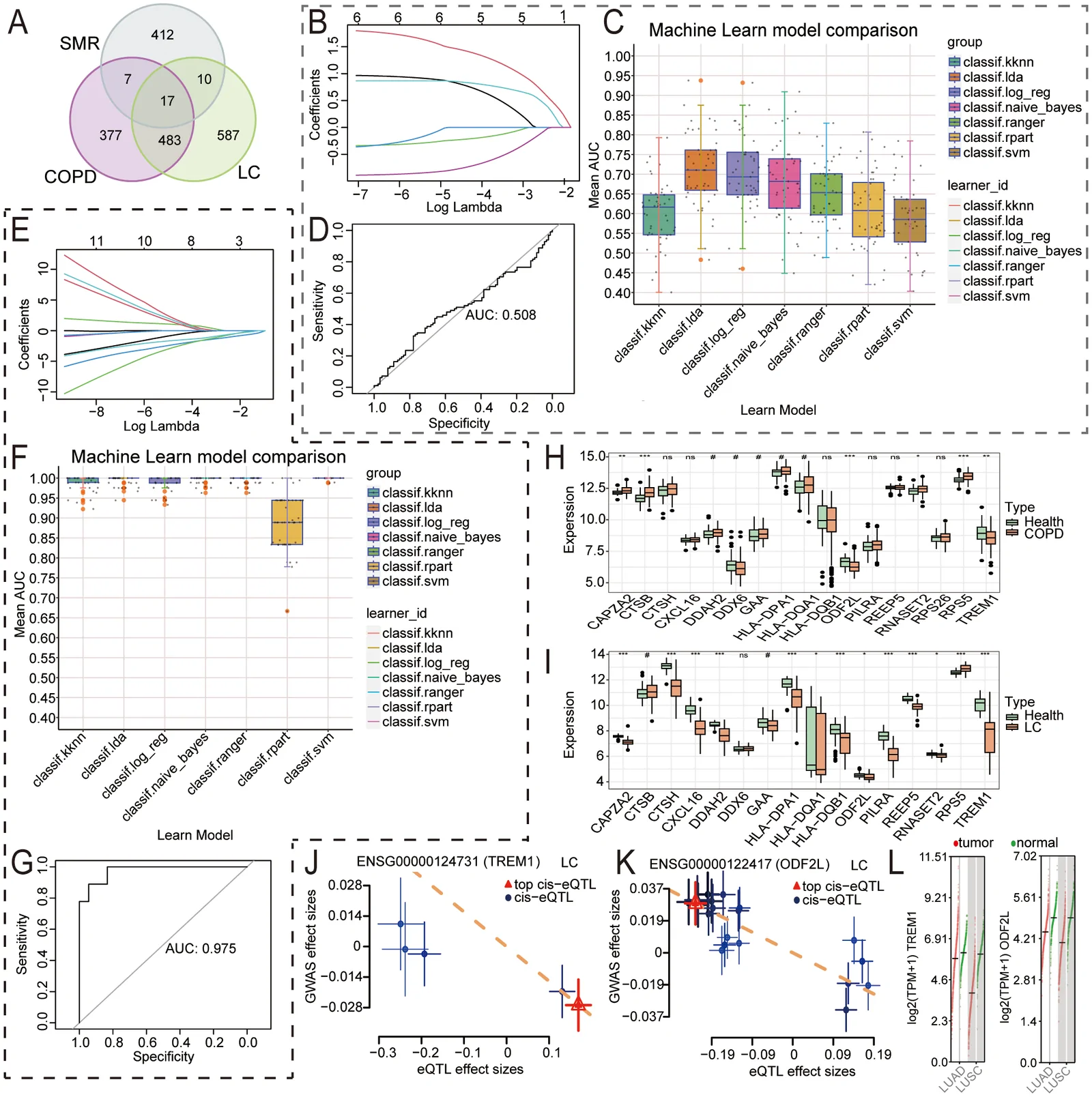
Integrative identification and validation of hub genes associated with COPD and LC. **(A)** Venn diagram showing the overlapping hub genes among SMR-identified genes, COPD-related genes, and LC-related genes. **(B–D)** Machine learning models for COPD diagnosis based on hub genes, including LASSO coefficient profiles **(B)**, comparison of model performance in the training set by mean AUC **(C)**, and the ROC curve in the test set **(D)**. **(E–G)** Machine learning models for LC diagnosis based on hub genes, including LASSO coefficient profiles **(E)**, comparison of model performance in the training set by mean AUC **(F)**, and the ROC curve in the test set **(G)**. **(H)** Expression levels of hub genes in healthy controls and COPD samples. **(I)** Expression levels of hub genes in healthy controls and LC samples. Statistical significance is indicated as ***P < 0.001, **P < 0.01, *P < 0.05, # 0.05 < P ≤ 0.2, and ns, not significant. **(J, K)** SMR plots showing the associations of TREM1 **(J)** and ODF2L **(K)** with LC using cis-eQTL instruments. **(L)** Differential expression of TREM1 and ODF2L between tumor and normal samples in the LUAD and LUSC cohorts based on GEPIA3.

To investigate the shared genetic and cellular mechanisms underlying COPD and LC, macrophage subpopulations within the pulmonary microenvironment were characterized. Macrophage-specific DEGs were extracted from scRNA-seq datasets of COPD (GSE173896) and LC (GSE117570), yielding 884 and 1,097 DEGs, respectively. Concurrently, SMR analysis was performed using summary statistics from an LC genome-wide association study (GWAS ID: ieu-a-987), identifying 446 instrumental variable genes with significant causal associations with LC (**Supplementary Table 5**). Intersection of these datasets yielded 17 overlapping genes (**Figure 5A**), which was subsequently narrowed to 14 genes after filtering by p_HEIDI > 0.05 (**Table 1**).

**Table 1:** Shared candidate genes for COPD and LC identified by SMR.

Chr	Gene	topSNP	A1	A2	b_GWAS	p_GWAS	b_eQTL	p_eQTL	b_SMR	p_SMR	p_HEIDI
5	REEP5	rs382427	G	A	0.0267	0.025	-0.1642	1.17×10^-^¹²	-0.1627	0.032	0.703
6	TREM1	rs6914090	C	T	-0.0269	0.022	0.1704	6.66×10^-9^	-0.1581	0.033	0.081
1	ODF2L	rs11161811	C	T	0.0289	0.019	-0.2253	1.08×10^-^²^0^	-0.1285	0.023	0.359
6	HLA-DQA1	rs9272320	A	G	0.0645	0.000	-0.6639	7.99×10^-89^	-0.0972	0.000	0.108
17	GAA	rs2304849	C	G	0.0394	0.006	-0.5653	1.51×10^-67^	-0.0698	0.006	0.363
6	HLA-DQB1	rs9273394	G	A	0.0560	0.000	-0.8232	7.82×10^-97^	-0.0680	0.000	0.994
12	RPS26	rs1131017	G	C	0.0306	0.010	-1.1089	0	-0.0276	0.010	0.054
7	PILRA	rs28495773	T	A	0.0332	0.030	0.3432	2.85×10^-^²²	0.0966	0.034	0.268
17	CXCL16	rs8071612	T	G	-0.0256	0.031	-0.1814	5.14×10^-^¹^7^	0.1408	0.037	0.326
6	RNASET2	rs398278	G	A	-0.0633	0.000	-0.3375	1.18×10^-^²^7^	0.1875	0.000	0.101
6	HLA-DPA1	rs116733694	G	A	-0.0507	0.004	-0.2268	2.67×10^-^¹^4^	0.2234	0.007	0.502
8	CTSB	rs1622599	G	A	-0.0274	0.025	-0.1212	2.26×10^-^¹²	0.2264	0.033	0.105
7	CAPZA2	rs12670264	C	G	-0.0394	0.004	-0.1629	4.29×10^-^¹^4^	0.2417	0.008	0.304
11	DDX6	rs470203	A	G	0.0422	0.001	0.1590	1.51×10^-8^	0.2652	0.005	0.285

COPD, chronic obstructive pulmonary disease; LC, lung cancer; SMR, summary data-based Mendelian randomization; SNP, single nucleotide polymorphism; eQTL, expression quantitative trait loci; HEIDI, heterogeneity in dependent instruments test; Chr, chromosome; A1, effect allele; A2, alternative allele; b, effect size; p, P-value.

The 14 candidate genes were subsequently used as input features for machine-learning analyses. LASSO regression was first applied for feature selection, after which the selected genes were incorporated into multiple classifiers for diagnostic model construction and evaluation. For COPD diagnosis, seven classifiers were trained using the GSE76925 dataset and subsequently evaluated in the independent validation cohort GSE57148 (**Figures 5B–D**). Although the models exhibited limited diagnostic performance for COPD, they enabled systematic evaluation of the candidate genes. For LC diagnosis, the same analytical strategy was applied using GSE18842 as the training dataset and GSE74706 as the independent validation dataset. Following LASSO feature selection (**Figure 5E**), evaluation in the training set indicated favorable model performance (**Figure 5F**), with the optimal model achieving an AUC of 0.975 in the validation cohort (**Figure 5G**).

Differential expression analysis in GSE76925 and GSE18842 revealed significant downregulation of TREM1 and ODF2L in both disease groups compared with controls (**Figures 5H, I**). SMR analysis further supported the associations of TREM1 and ODF2L with LC susceptibility (**Figures 5J, K**), and GEPIA3 database analysis confirmed their downregulation across LC histological subtypes (**Figure 5L**). To further characterize the macrophage populations associated with these hub genes, macrophage subpopulations were annotated using established marker genes as inflammatory macrophages, resident alveolar macrophages, monocyte-derived macrophages, and tumor-associated macrophages. The marker-expression profiles used for subtype annotation are presented in **Supplementary Figure 4A** (COPD) and **Supplementary Figure 4E** (LC), whereas the corresponding subtype annotations are shown in **Supplementary Figure 4B** and S4F, respectively. In the COPD dataset, TREM1 was predominantly enriched in inflammatory macrophages and exhibited broader expression in COPD tissues than in normal controls, whereas ODF2L showed relatively low and diffuse expression across macrophage subsets (**Supplementary Figures 4C, D**). Similarly, in the LC dataset, TREM1 was mainly enriched in inflammatory macrophages and tumor-associated macrophages, with stronger expression in tumor tissues than in normal controls, whereas ODF2L remained weakly expressed without obvious subtype specificity (**Supplementary Figures 4G, H**). Based on the computational prioritization and external validation results, TREM1 and ODF2L were selected for subsequent experimental validation.

### 3.5 Validation of TREM1 and ODF2L expression in lung cell lines and tissues

**Figure 6:**
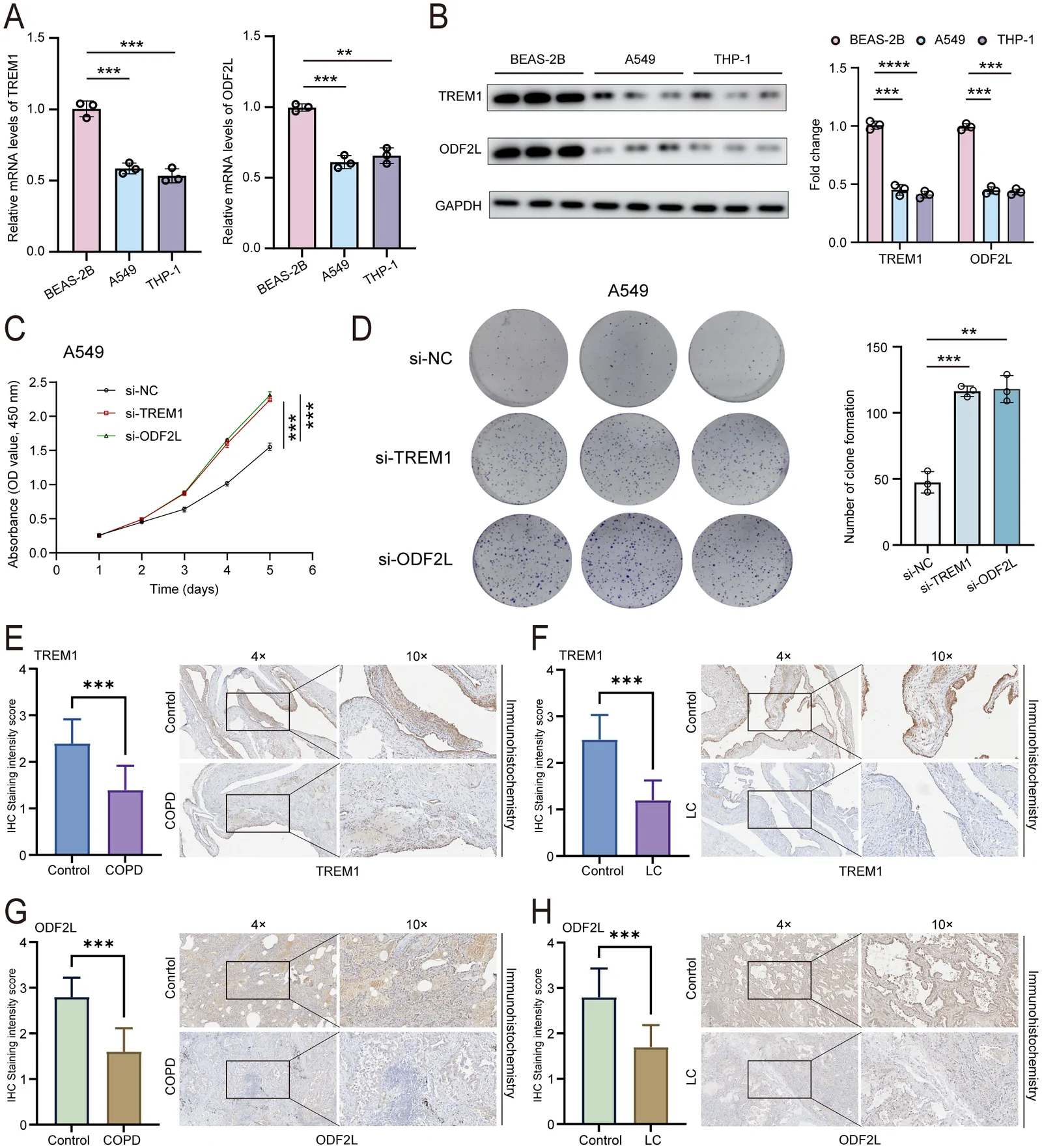
Validation of TREM1 and ODF2L expression in COPD and lung cancer tissues. **(A)** Relative mRNA levels of TREM1 and ODF2L in BEAS-2B, A549, and THP-1 as determined by RT-qPCR. **(B)** Western blot analysis (left) and quantitative densitometry (right) of TREM1 and ODF2L protein expression in BEAS-2B, A549, and THP-1 cells. **(C)** Cell proliferation curves of A549 cells transfected with si-NC, si-TREM1, or si-ODF2L as measured by CCK-8 assay. **(D)** Representative images (left) and quantification (right) of colony formation assays in A549 cells following knockdown of TREM1 or ODF2L. **(E–H)** IHC staining intensity scores and representative images of TREM1 **(E, F)** and ODF2L **(G, H)** in control, COPD, and LC tissue sections. Statistical significance is indicated as ****P < 0.0001, ***P < 0.001, and **P < 0.01.

The expression patterns of TREM1 and ODF2L were validated in lung cell lines and tissue samples using RT-qPCR, western blotting, and immunohistochemistry. RT-qPCR analysis showed that TREM1 mRNA expression was significantly reduced in both A549 and THP-1 cells compared with BEAS-2B cells (both P < 0.001), whereas ODF2L mRNA expression was significantly decreased in A549 (P < 0.01) and THP-1 (P < 0.001) cells (**Figure 6A**). Consistent with the mRNA results, western blot analysis demonstrated markedly lower protein expression levels of TREM1 and ODF2L in A549 and THP-1 cells than in BEAS-2B cells (both P < 0.001) (**Figure 6B**). Loss-of-function experiments were subsequently performed in A549 cells. CCK-8 assays showed that silencing either TREM1 or ODF2L significantly increased cell proliferative capacity compared with the negative control (both P < 0.001) (**Figure 6C**). Similarly, colony formation assays demonstrated significantly increased colony numbers following knockdown of TREM1 (P < 0.001) or ODF2L (P < 0.01) (**Figure 6D**), indicating that reduced expression of either gene promotes the growth of lung cancer cells. The expression of TREM1 and ODF2L was further examined in clinical tissue specimens by immunohistochemistry. TREM1 immunoreactivity was markedly decreased in both COPD and LC tissues compared with normal lung tissues (both P < 0.001) (**Figures 6E, F**). Likewise, ODF2L staining intensity was significantly lower in COPD and LC tissues than in normal controls (both P < 0.001) (**Figures 6G, H**).

### 3.6 Macrophage-based functional validation of TREM1 and ODF2L

**Figure 7:**
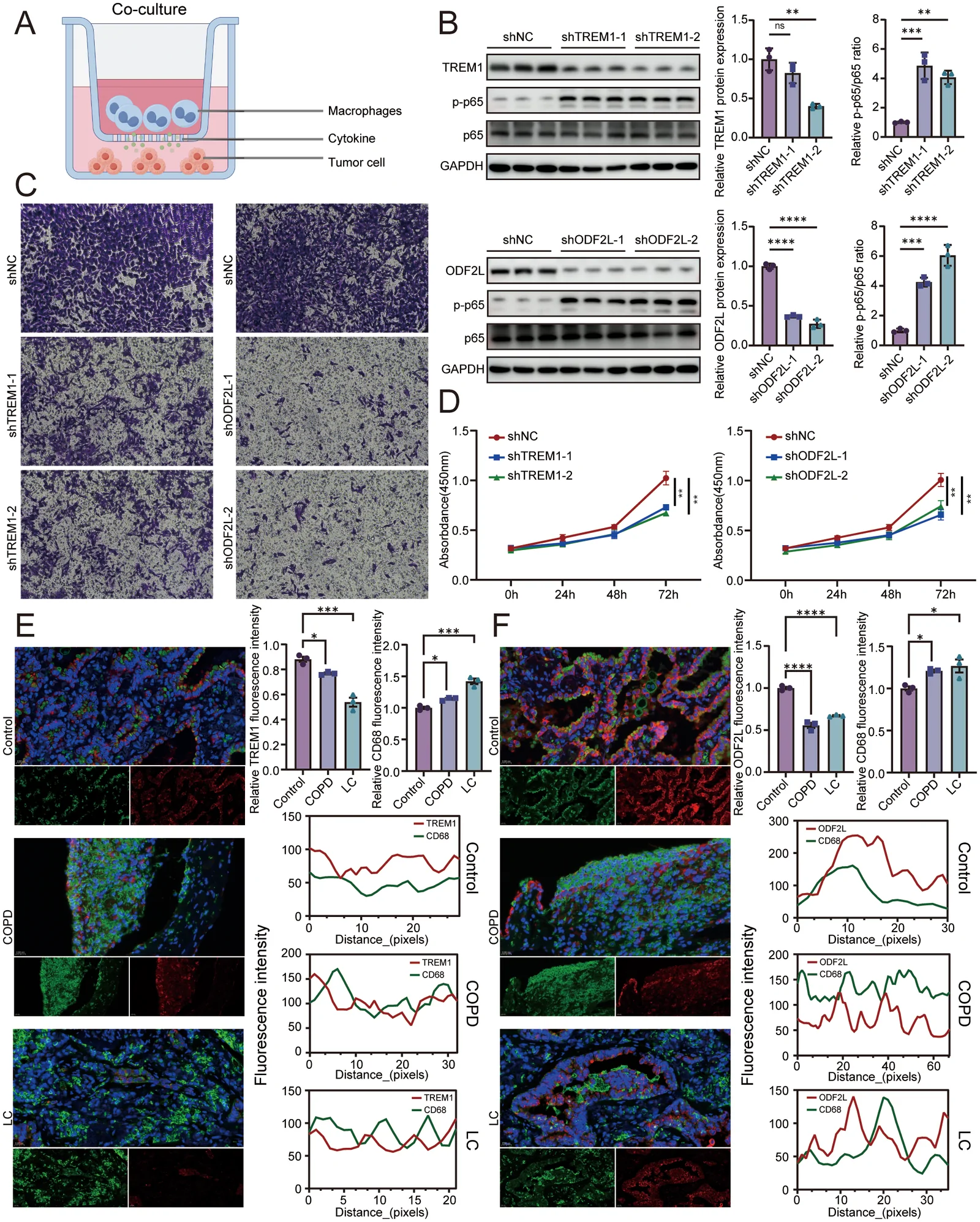
Macrophage-based functional validation and cellular localization of TREM1 and ODF2L. **(A)** Schematic illustration of the indirect Transwell co-culture system between THP-1-derived macrophages and A549 cells used for macrophage-based functional validation. **(B)** Representative western blot images and densitometric analysis showing the knockdown efficiency of TREM1 and ODF2L in THP-1-derived macrophages, together with the corresponding expression levels of phosphorylated NF-κB p65 (p-p65) and total p65. **(C)** Representative images of Transwell migration assays showing the effects of macrophage-specific knockdown of TREM1 or ODF2L on the migratory ability of A549 cells. **(D)** CCK-8 assays showing the effects of macrophage-specific knockdown of TREM1 or ODF2L on A549 cell proliferation. **(E)** Representative merged and single-channel dual immunofluorescence images of TREM1 (red) and the macrophage marker CD68 (green) in normal lung, COPD, and LC tissues, with nuclei counterstained with DAPI (blue). Quantitative analysis of the relative fluorescence intensities of TREM1 and CD68, together with line profile analysis of TREM1 and CD68 fluorescence signals along the selected regions of interest, are shown. Original magnification, ×40. **(F)** Representative merged and single-channel dual immunofluorescence images of ODF2L (red) and the macrophage marker CD68 (green) in normal lung, COPD, and LC tissues, with nuclei counterstained with DAPI (blue). Quantitative analysis of the relative fluorescence intensities of ODF2L and CD68, together with line profile analysis of ODF2L and CD68 fluorescence signals along the selected regions of interest, are shown. Original magnification, ×40. Original magnification, ×40. Statistical significance is indicated as ****P < 0.0001, ***P < 0.001, **P < 0.01, and *P < 0.05.

THP-1-derived macrophages were transfected with two independent shRNAs targeting TREM1 or ODF2L, followed by indirect Transwell co-culture with A549 cells (**Figure 7A**). Western blot analysis confirmed efficient knockdown of both genes in THP-1-derived macrophages. Silencing either TREM1 or ODF2L increased the phosphorylation level of NF-κB p65, whereas the total p65 protein level remained largely unchanged (**Figure 7B**). In the indirect co-culture system, macrophage-specific knockdown of either gene significantly attenuated the migratory ability of A549 cells, as demonstrated by the Transwell migration assay (**Figure 7C**). Consistently, CCK-8 assays showed that A549 cells co-cultured with TREM1- or ODF2L-silenced macrophages exhibited significantly reduced proliferative capacity compared with the control group (**Figure 7D**).

Dual immunofluorescence staining demonstrated that both TREM1 and ODF2L were partially co-localized with the macrophage marker CD68 in lung tissues (**Figures 7E, F**). Quantitative fluorescence analysis revealed significantly lower fluorescence intensities of TREM1 and ODF2L in COPD and LC tissues than in normal lung tissues. In contrast, CD68 fluorescence intensity was significantly increased in both COPD and LC tissues compared with normal lung tissues (**Figures 7E, F**). Furthermore, line profile analysis demonstrated partial spatial overlap between TREM1 and CD68, as well as between ODF2L and CD68, within representative regions of interest, supporting the macrophage-associated localization of both proteins (**Figures 7E, F**).

## Discussion

This study characterized the comorbidity between COPD and LC by integrating global epidemiological datasets with multi-omics analyses, identifying a geographically consistent pattern of co-occurrence that persisted over the study period and that may reflect shared environmental exposures and biological mechanisms. These observations are consistent with prior evidence that COPD is associated with an increased risk of LC and that chronic inflammation constitutes a plausible common pathway linking the two conditions (7), and they accord with recent syntheses implicating genomic, immune and microenvironmental dysregulation in COPD–LC comorbidity (42). At the exposure level, modelling identified tobacco smoke, ambient particulate pollution and radon as important contributors to joint burden, while inclusion of climatic variables indicated that non-traditional exposures merit further evaluation. Integrative transcriptomic and single-cell analyses identified concordant alterations in immune cell populations, notably macrophage-related signatures, across COPD and LC samples, which provide additional, indirect molecular support for inflammation-related links between the conditions. Crucially, our integrative pipeline identified TREM1 and ODF2L as shared hub genes, and subsequent validation in lung cell lines, THP-1-derived macrophages, and clinical tissues provided experimental support for their functional relevance. Macrophage-specific silencing further altered macrophage-mediated regulation of A549 cell migration and proliferation, while dual immunofluorescence demonstrated the macrophage-associated localization of TREM1 and ODF2L in lung tissues. Collectively, these findings provide complementary epidemiological, environmental, transcriptomic, genetic, and experimental evidence supporting the hypothesis that COPD and LC share common pathogenic mechanisms. By integrating these complementary levels of evidence, our study progressively narrowed the investigation from population-level disease patterns to macrophage-associated molecular candidates and their biological validation, providing a more comprehensive framework for understanding COPD–LC comorbidity.

Building on these overall observations, this study extends existing epidemiological and exposure-related knowledge of COPD–LC comorbidity by confirming that tobacco smoking remains the principal shared risk factor and by implicating ambient particulate matter, particularly PM2.5, and occupational exposures as important contributors to disease occurrence and progression (43, 44). Evidence from recent work further indicates that particulate pollution may contribute to lung carcinogenesis through pathways related to inflammation, oxidative stress, and immunomodulation (45). Indoor exposures, notably residential radon and other forms of household pollution, have been associated with elevated LC risk, particularly among never-smokers, and warrant consideration in risk appraisals (46, 47). Consistent with these reports, our modelling identified smoking, particulate pollution and radon as leading contributors to joint disease burden, while application of SHAP analysis provided quantitative estimates of their relative contributions across comorbidity patterns; moreover, integration of spatial distributions with a multidimensional exposome perspective revealed heterogeneity in regional risk-factor combinations, underscoring the value of moving from single-exposure or single-disease analyses toward a comorbidity-oriented, multi-exposure framework to better capture the cooperative effects of environmental hazards on chronic respiratory disease.

Complementing these exposure-level findings, our multi-omics analyses support chronic inflammation and remodeling of the pulmonary immune microenvironment as a plausible mechanistic pathway linking COPD and LC. Previous studies have characterized COPD as a condition of persistent airway inflammation that can persist after smoking cessation and involve sustained activity of inflammatory mediators (e.g., TNF-α, IL-6) and reactive oxygen species, changes that have been associated with genomic instability and tumorigenesis (48, 49). Macrophages appear to be central effectors in this context: chronic inflammatory stimuli promote macrophage recruitment and activation, whereas the tumor microenvironment can induce macrophage polarization toward immunosuppressive (M2-like) phenotypes that support tumor growth and inhibit anti-tumor immunity (12, 50). Inflammatory signaling pathways such as NF-κB have been implicated in sustaining an immune milieu marked by increased M2 macrophages and regulatory T cells, consistent with a shift toward immunosuppression that may act in concert with inflammation to facilitate tumorigenesis (12). Our single-cell analyses reveal concordant alterations in macrophage-related subsets across COPD and LC samples, consistent with prior observations of increased macrophage infiltration and immune remodeling in tumors from patients with COPD (51). Furthermore, macrophage-based functional experiments demonstrated that silencing TREM1 or ODF2L in THP-1-derived macrophages altered macrophage-mediated regulation of A549 cell migration and proliferation, providing additional experimental support for the involvement of macrophages in COPD–LC-associated immune remodeling. Overall, these observations indicate that COPD-associated chronic inflammation and macrophage-centered immune remodeling plausibly contribute to the epidemiological association between COPD and LC, while acknowledging that genetic susceptibility and heterogeneous exposure histories are additional determinants of disease heterogeneity.

Extending this immune-microenvironment perspective to the molecular level, our multi-omics integration implicates inflammatory signaling and immune microenvironment remodeling as plausible mediators linking chronic obstructive pulmonary disease and LC, and identifies TREM1 and ODF2L as candidate nodes within this network. TREM1 is a myeloid-expressed receptor that amplifies innate inflammatory responses and has been implicated in the modulation of macrophage and myeloid-derived suppressor cell functions across inflammatory and neoplastic contexts; its activity is mechanistically connected to canonical inflammatory effectors including NF-κB and downstream cytokine signaling, which can reinforce an immunosuppressive, tumor-permissive microenvironment (52). Previous evidence has demonstrated that TREM1 is a key amplifier of innate immune inflammatory signaling (53). In multiple malignancies, TREM1-mediated inflammatory responses have been shown to promote a pro-tumor inflammatory microenvironment. However, its cellular distribution and functional contribution appear to vary across tumor types. In inflammation-driven intestinal tumorigenesis, for example, TREM1 was primarily detected in tumor-infiltrating neutrophils rather than tumor-associated macrophages, suggesting that its biological effects depend on the cellular and tissue context of tumor-associated inflammation (53, 54). Our observation that TREM1 silencing altered NF-κB p65 phosphorylation in THP-1-derived macrophages further supports its involvement in macrophage-associated inflammatory regulation, although the underlying mechanisms require further investigation. In addition, dual immunofluorescence demonstrated partial spatial overlap between TREM1 and CD68-positive macrophages in human lung tissues, providing tissue-level evidence that TREM1 is associated with macrophage populations within the COPD–LC microenvironment. This observation is consistent with our finding that TREM1 is associated with, but not restricted to, macrophages within the COPD–LC microenvironment. ODF2L is less well characterized in pulmonary disease, but recent functional studies identify ODF2L as a centrosome/cilia-related protein with roles in epithelial structural biology and, in cancer models, as a modulator of cell cycle checkpoint dependencies; the consistent expression changes and causal-inference signals observed in our analyses nominate ODF2L as a previously underappreciated molecular connector between epithelial integrity and inflammation-associated tumor risk (55). Our functional experiments demonstrated that silencing TREM1 or ODF2L produced distinct biological effects in epithelial cells and macrophages. Specifically, knockdown of either gene promoted A549 cell proliferation, whereas macrophage-specific silencing attenuated the ability of THP-1-derived macrophages to promote A549 cell migration and proliferation. These findings suggest that the biological functions of TREM1 and ODF2L are likely to be cell type-dependent rather than uniformly tumor suppressive or tumor promoting. The proliferative phenotype following knockdown in A549 cells is consistent with a cell-intrinsic growth-restraining function in this epithelial cancer cell model, whereas the macrophage knockdown and co-culture findings support additional roles in macrophage-associated immune regulation and macrophage–epithelial communication. Accordingly, the present evidence does not support classifying TREM1 or ODF2L exclusively as epithelial tumor suppressors or tumor-promoting immune regulators; rather, their functions appear to depend on the cellular compartment and microenvironmental context. It should be noted that the prioritization of TREM1 and ODF2L did not rely solely on their diagnostic performance in the machine-learning models. Instead, these genes were consistently supported by multiple complementary analytical layers, including macrophage-specific differential expression, SMR analysis, feature selection, independent machine-learning evaluation, and subsequent experimental validation. Notably, the LC model achieved substantially higher diagnostic performance than the COPD model. This difference may partly reflect the use of LC GWAS summary statistics during the SMR prioritization step, which could preferentially enrich genes with stronger genetic relevance to lung cancer. Therefore, TREM1 and ODF2L are more appropriately interpreted as shared macrophage-associated candidate genes linking COPD and LC, while exhibiting greater diagnostic relevance in lung cancer than in COPD. An apparent discrepancy was observed between the bulk transcriptomic and single-cell analyses. While bulk transcriptomic analysis demonstrated reduced overall expression of TREM1 and ODF2L in COPD and LC tissues, single-cell analysis showed that TREM1 expression was preferentially enriched in inflammatory macrophages in COPD and in inflammatory as well as tumor-associated macrophages in LC, with broader distribution in diseased tissues than in controls. These findings are not necessarily contradictory because bulk RNA sequencing quantifies the average transcript abundance across all cell populations within a tissue, whereas single-cell RNA sequencing resolves cell type-specific expression and changes in cellular composition (56). Therefore, selective expansion of inflammatory and tumor-associated macrophage subsets may increase the proportion of TREM1-expressing macrophages despite an overall reduction in tissue-level expression. In addition, disease-associated alterations in non-macrophage cell populations, together with cell type-specific transcriptional regulation, may further contribute to the reduced bulk expression of TREM1 and ODF2L (57). Similar differences between tissue-level and cell type-specific transcriptional patterns have also been reported in single-cell analyses of COPD lungs. The partial spatial overlap between TREM1 and CD68 observed by dual immunofluorescence is consistent with these findings and further supports the value of integrating bulk transcriptomic, single-cell, and tissue-level analyses when interpreting disease-associated molecular alterations. These convergent computational and experimental observations nominate TREM1 and ODF2L as functionally relevant molecules in COPD–LC comorbidity, although the precise mechanisms by which they modulate macrophage function and inflammatory signaling in this context remain to be fully elucidated and warrant further investigation.

Methodologically, these convergent epidemiological, exposure-related, cellular, and molecular inferences are supported by several complementary design features that increase the interpretability of the findings while acknowledging inherent constraints. Use of Global Burden of Disease data allowed systematic mapping of spatial concordance in COPD and LC burden at a global scale, providing an empiric basis for examining shared exposures and regional heterogeneity. Integration of bulk transcriptomic and single-cell datasets with causal-inference approaches and targeted machine-learning explainability (for example SHAP) enabled interrogation of mechanistic signals across organizational scales, from population-level exposure patterns to cell-type specific transcriptional programs. Specifically, SHAP facilitated the interpretation of major exposure-related risk factors, SMR provided genetic evidence for candidate susceptibility genes, and single-cell analysis characterized their expression within specific cellular populations. Together, these approaches provided complementary epidemiological, genetic, and cellular perspectives for interpreting COPD–LC comorbidity. This multi-scale strategy is further strengthened by the integration of *in vitro* functional validation in both lung epithelial cells and THP-1-derived macrophages, indirect macrophage–epithelial Transwell co-culture assays, immunohistochemical analysis, and dual immunofluorescence staining in clinical specimens, thereby providing complementary experimental support for the computationally prioritized candidate genes (42). Cross-validation across independent datasets strengthened robustness, and the concordance between in silico predictions and experimental results supports the reliability of the identified hub genes. Nevertheless, the ecological nature of global burden data, heterogeneity in publicly available omics sources, and the assumptions required for genetic instrumental variable methods warrant caution in causal interpretation. Although the present study incorporated functional validation in both epithelial cells and macrophages, together with tissue-level localization analyses, the molecular mechanisms through which TREM1 and ODF2L regulate macrophage-associated immune remodeling and macrophage–epithelial communication remain to be fully elucidated. Furthermore, the indirect co-culture system employed here cannot fully recapitulate the complexity of the *in vivo* pulmonary immune microenvironment, and additional mechanistic studies are warranted to clarify the downstream signaling events involved.

Taken together, these epidemiological, environmental, and molecular findings have implications for both population-level prevention and clinical practice. Identification of shared, modifiable exposures—notably tobacco smoking, ambient fine particulate pollution and residential radon—reinforces the rationale for strengthened tobacco-control policies, measures to reduce population exposure to PM2.5, and targeted radon mitigation in high-exposure dwellings (44, 46). From a clinical perspective, COPD identifies a patient population at elevated risk of LC for whom risk-stratified surveillance and intensified case-finding merit consideration; the integrative analyses and experimental validation performed in this study further suggest that TREM1- and ODF2L-associated molecular programs, particularly those related to macrophage-associated immune regulation, may represent promising candidate biomarkers and potential therapeutic targets. However, given the cell type-dependent effects observed in epithelial cells and macrophages, their clinical applicability requires further validation in prospective cohorts and mechanistic studies before translational implementation (58). The multi-exposure, comorbidity-oriented analytic framework employed here further suggests that integrated risk assessment and coordinated preventive strategies addressing shared determinants may be more effective than isolated, disease-specific approaches, although policy and practice recommendations should await confirmatory evidence.

At the same time, several important limitations qualify these conclusions and indicate priorities for future work. Analyses based on aggregated Global Burden of Disease data are ecological and do not permit direct inference of individual-level causation; consequently, the observed spatial concordance and comorbidity patterns should not be interpreted as establishing that individual exposure–disease relationships exist in the same form at the population level. The multi-omic datasets used for mechanistic inference were drawn from heterogeneous public repositories and vary in cohort composition, sampling procedures, and assay platforms, which may affect effect estimates and limit generalizability. Additionally, validation using GEPIA3 relied on TCGA RNA-seq data, which differ from the GEO datasets used elsewhere in cohort composition, sample processing, and sequencing platforms; this clinical and technical heterogeneity may affect cross-database comparability and should be considered when interpreting the expression patterns. Causal-inference approaches applied in this study, including summary-data Mendelian randomization, depend on instrument validity and on assumptions that cannot replace randomized or mechanistic experimental evidence. Although functional validation of TREM1 and ODF2L was performed *in vitro*, the precise molecular mechanisms through which these genes regulate macrophage-associated immune remodeling remain to be fully elucidated (42). Finally, uneven regional data availability and variable data quality may influence global maps of co-occurrence. Taken together, these constraints indicate that prospective, individual-level epidemiological studies and additional mechanistic investigations are necessary to validate the hypotheses generated and to assess their translational potential.

## Conclusion

In conclusion, this study systematically characterized the comorbidity between COPD and LC using an integrative framework incorporating global epidemiological analysis, environmental exposure assessment, multi-omics profiling, and artificial intelligence–based modeling. The results suggest that these two diseases may share common patterns at both the population and biological levels, involving environmental factors, particularly smoking, particulate matter pollution, and residential radon, as well as alterations in the immune microenvironment, especially macrophage-related changes. In addition, TREM1 and ODF2L were identified and experimentally validated as shared hub genes, with functional studies supporting their context-dependent roles in COPD–LC comorbidity. Collectively, these findings provide a multi-scale perspective for understanding the relationship between COPD and LC. Further mechanistic and longitudinal studies are warranted to clarify causal relationships and support clinical translation.

## Data Availability

The original contributions presented in the study are included in the article/**Supplementary Material**. Further inquiries can be directed to the corresponding authors.

## Supplementary material

Supplementary Figure 1 Global epidemiological patterns, temporal trends, and inter-country similarity network of COPD and LC. **(A, B)** Global distribution of the age-standardized incidence rate (ASIR) of COPD **(A)** and LC **(B)** in 2021. **(C–F)** Annual changes in the comorbidity severity score and comorbidity index of COPD and LC in developed countries **(C)**, emerging economies **(D)**, least developed countries **(E)**, and other developing countries **(F)**. Colors indicate the comorbidity severity score, and dot size represents the comorbidity index. **(G)** Network matrix showing inter-country similarity patterns. Both axes represent countries ordered by community structure. Colors indicate the degree of network similarity, symbols denote connection type, and dot size indicates connection strength.

Supplementary Figure 2 SHAP-based identification of major risk factors associated with different COPD–LC comorbidity patterns and their global exposure distributions. **(A–C)** SHAP summary plots showing the relative contributions of risk factors in the consistent **(A)**, COPD-dominant **(B)**, and LC-dominant **(C)** patterns. Each point represents one country or region; the x-axis indicates the SHAP value, and color denotes the feature value from low to high. **(D)** Global distribution of exposure rates for the identified risk factors.

Supplementary Figure 3 Quality control and cell-type annotation of scRNA-seq data. **(A, B)** Quality control metrics for GSE173896 (COPD) and GSE117570 (LC), including nFeature_RNA, nCount_RNA, percent.mt, and percent.rb. **(C, D)** UMAP plots showing cell clustering in normal and disease samples. **(E, F)** Heatmaps of marker gene expression across cell clusters used for cell-type annotation.

Supplementary Figure 4 Annotation of macrophage subpopulations and expression patterns of TREM1 and ODF2L in COPD and LC single-cell datasets. **(A)** Heatmap showing the expression of representative marker genes used for macrophage subpopulation annotation in the COPD dataset (GSE173896). **(B)** UMAP visualization of annotated macrophage subpopulations in normal and COPD tissues. **(C, D)** UMAP feature plots showing the expression of TREM1 and ODF2L in macrophage subpopulations from normal **(C)** and COPD **(D)** tissues. **(E)** Heatmap showing the expression of representative marker genes used for macrophage subpopulation annotation in the LC dataset (GSE117570). **(F)** UMAP visualization of annotated macrophage subpopulations in normal and LC tissues. **(G, H)** UMAP feature plots showing the expression of TREM1 and ODF2L in macrophage subpopulations from normal **(G)** and LC **(H)** tissues.

Supplementary Table 5 Full results of the SMR analysis for lung cancer using cis-eQTL data.
