# Supplementary material for: SHAP-interpretable machine learning integrating exposures and multi-omics reveals immune alterations and biomarkers in COPD–lung cancer comorbidity
Source: Front Immunol. 2026 Aug 7;17:1875146. doi: 10.3389/fimmu.2026.1875146 (PMC13494581; doi:10.3389/fimmu.2026.1875146)
Supplement: Supplementary file 1 [file Table1.docx]

Supplementary Table 1. Primers used for quantitative real-time PCR.

| Gene | Forward (5′→3′) | Reverse (5′→3′) |
| --- | --- | --- |
| TREM1 | GAACTCCGAGCTGCAACTAAA | TCTAGCGTGTAGTCACATTTCAC |
| ODF2L | AAAGCAAACCGTTTTTCCCAATC | CGTTCTCGGCTTCCCTTTTATG |
| GAPDH | CACAAGCAGAGTGCTGAAGGTG | ACCACCCTGTTGCTGTAGCCAA |
