# Supplementary material for: SHAP-interpretable machine learning integrating exposures and multi-omics reveals immune alterations and biomarkers in COPD–lung cancer comorbidity
Source: Front Immunol. 2026 Aug 7;17:1875146. doi: 10.3389/fimmu.2026.1875146 (PMC13494581; doi:10.3389/fimmu.2026.1875146)
Supplement: Supplementary file 2 [file Table2.docx]

Supplementary Table 2. Antibodies used for Western blotting, immunohistochemistry (IHC), and immunofluorescence (IF).

| Antibody Name | Host | Application | Dilution | Catalog No. | Source |
| --- | --- | --- | --- | --- | --- |
| TREM1 Polyclonal Antibody | Rabbit | WB, IHC, IF | 1:1000 | HV219014 | abinScience, China |
| ODF2L Polyclonal Antibody | Rabbit | WB, IHC, IF | 1:1000 | YN3657 | Immunoway, China |
| Phospho-NF-κB p65 (Ser536) Monoclonal Antibody | Rabbit | WB | 1:1000 | 3033 | Cell Signaling Technology, USA |
| NF-κB p65 Monoclonal Antibody | Rabbit | WB | 1:1000 | 8242 | Cell Signaling Technology, USA |
| CD68 Monoclonal Antibody (Clone KP1) | Mouse | IF | 1:200 | ab955 | Abcam, UK |
| GAPDH Monoclonal Antibody | Mouse | WB | 1:5000 | 60004-1-Ig | Proteintech, China |
| HRP‑conjugated Goat Anti-Rabbit IgG (H+L) | Goat | WB | 1:5000 | SA00001-2 | Proteintech, China |
| HRP‑conjugated Goat Anti-Mouse IgG (H+L) | Goat | WB | 1:5000 | SA00001-1 | Proteintech, China |
| Alexa Fluor® 594 Goat Anti-Rabbit IgG (H+L) | Goat | IF | 1:500 | A-11012 | Invitrogen, USA |
| Alexa Fluor™ 488 Goat Anti-Mouse IgG (H+L) | Goat | IF | 1:500 | A-11001 | Invitrogen, USA |
