# Supplementary material for: SHAP-interpretable machine learning integrating exposures and multi-omics reveals immune alterations and biomarkers in COPD–lung cancer comorbidity
Source: Front Immunol. 2026 Aug 7;17:1875146. doi: 10.3389/fimmu.2026.1875146 (PMC13494581; doi:10.3389/fimmu.2026.1875146)
Supplement: Supplementary file 3 [file Table3.docx]

Supplementary Table 3. Oligonucleotide sequences used for gene knockdown.

| siRNA name | sense（5'-3'） | | antisense（5'-3'） |  |
| --- | --- | --- | --- | --- |
| Negative control | UUCUCCGAACGUGUCACGUTT | | ACGUGACACGUUCGGAGAATT |  |
| siRNA-TREM1 | CCGGUGUUCAAUAUUGUCAUU | | AAUGAUAAUGUUGAACACCGG |  |
| siRNA-ODF2L | CCAGTGAAAGUCAUCUCAGCU | | AGCUGAGAUGACUUUCACUGG |  |
|  |  | |  |  |
| shRNA | | Target sequence (5'-3') | | |
| shNC | | TTCTCCGAACGTGTCACGT | | |
| shTREM1-1 | | AGCCAGAAAGCTTGGCAGATA | | |
| shTREM1-2 | | GAGGATCATACTAGAAGACTA | | |
| shODF2L-1 | | CCAGTGAAAGTCATCTCAGCT | | |
| shODF2L-2 | | GCGGAGTTGGTAACTCATTCT | | |
