# Supplementary material for: SHAP-interpretable machine learning integrating exposures and multi-omics reveals immune alterations and biomarkers in COPD–lung cancer comorbidity
Source: Front Immunol. 2026 Aug 7;17:1875146. doi: 10.3389/fimmu.2026.1875146 (PMC13494581; doi:10.3389/fimmu.2026.1875146)
Supplement: Supplementary file 4 [file Table4.docx]

Supplementary Table 4. Marker genes used for cell-type annotation in the COPD and LC single-cell RNA sequencing datasets.

| **Cell type** | **COPD dataset (GSE173896)** | **LC dataset (GSE117570)** |
| --- | --- | --- |
| T cells | CD3D, CD3E, TRAC, TRBC2 | IL7R, SARAF, RPS29 |
| NK cells | GNLY, NKG7, KLRD1, CCL4 | CD69, HLA-E, HLA-A, HLA-B, B2M, FCGR3A |
| Macrophages | C1QA, C1QB, MRC1, CD68, MSR1 | CD68, CTSD, ACP5, APOC1, DAB2,CD206 |
| Inflammatory monocytes | S100A8, S100A9, S100A12, CXCL8 | FCN1, VCAN, IL1B, EREG, BCL2A1, PLAUR, SOD2 |
| Endothelial cells | EPAS1, EMCN, CLDN5, AQP1, RAMP2, GNG11 | — |
| Fibroblasts | LUM, DCN, MFAP4, MGP, CFD | — |
| Alveolar type II (AT2) cells | SFTPB, SLC34A2, SFTA2 | — |
| Smooth muscle cells | TAGLN, TPM2, MYL9, ACTA2, MYH11, PDGFRB | — |
| Mast cells | TPSB2, TPSAB1, CPA3, MS4A2, HPGDS, KIT, SLC18A2 | — |
| B cells | MS4A1, BANK1, CD79B, TNFRSF13C | CD37, RPS8 |
| Plasma cells | IGHG1, IGHG2, MZB1 | SEC11C, PRDX4, SSR4, HERPUD1, FKBP2, XBP1, SSR3, CYBA |
| Ciliated epithelial cells | TPPP3, RSPH1, CETN2 | — |
| Epithelial cells | — | SLC34A2, ELF3, KRT8 |
| Dendritic cells | — | HLA-DPB1, HLA-DQA1, HLA-DPA1, HLA-DQB1, HLA-DRA, HLA-DRB1, CD74 |
